# Faggot cells in therapy‐related acute myeloid leukemia with inv(16)

**DOI:** 10.1002/ccr3.3462

**Published:** 2020-11-22

**Authors:** Ana Vega González de Viñaspre, Carlos De Miguel Sánchez, Diego Robles De Castro, Verónica Roldán Galiacho, Arantza Mendizabal Abad, José María Guinea De Castro

**Affiliations:** ^1^ Hematology Division Hospital Universitario de Álava Vitoria‐Gasteiz Spain; ^2^ Hematology Division Hospital Universitario Cruces Bilbao Spain

**Keywords:** faggot cells, faggot neutrophils, therapy‐related acute myeloid leukemia (t‐AML) with inv(16)

## Abstract

Faggot cells are an uncommon finding in nonacute promyelocytic leukemia, even rarer when observed in mature granulocytic cells. Inv(16) should be dismissed when pre‐eosinophilic granulation and faggot neutrophils are observed.

## Graphical abstract

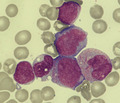

A 49‐year‐old woman presented with epistaxis. Her past medical history included breast carcinoma, which was treated 2 years ago with chemotherapy (including doxorubicin) achieving complete remission. On admission, a blood count revealed: hemoglobin 101 g/L, leukocyte count 123 × 10^9^/L, and platelets 5 × 10^9^/L. Peripheral blood smear showed 69% of agranular blast cells. Auer rods were not observed.

Bone marrow (BM) aspirate showed an hypercellular marrow with 59% of blast cells with myeloid (52%) and monocytoid (7%) appearance (Figure 1A). Isolated Auer rods (rod‐shaped cytoplasmic inclusions resulting from the crystallization of azurophilic granules) and pseudo‐Chediak‐Higashi granules were observed in myeloid blasts and promyelocytes, respectively (Figure 1B). Maturing granulocytic cells showed dysplastic features, highlighting the presence of bundles of Auer rods (faggot cells) from promyelocytes to mature neutrophils (Figure 1C). Eosinophils and its precursors showed pre‐eosinophilic granulation (Figure 1D).

**FIGURE 1:**
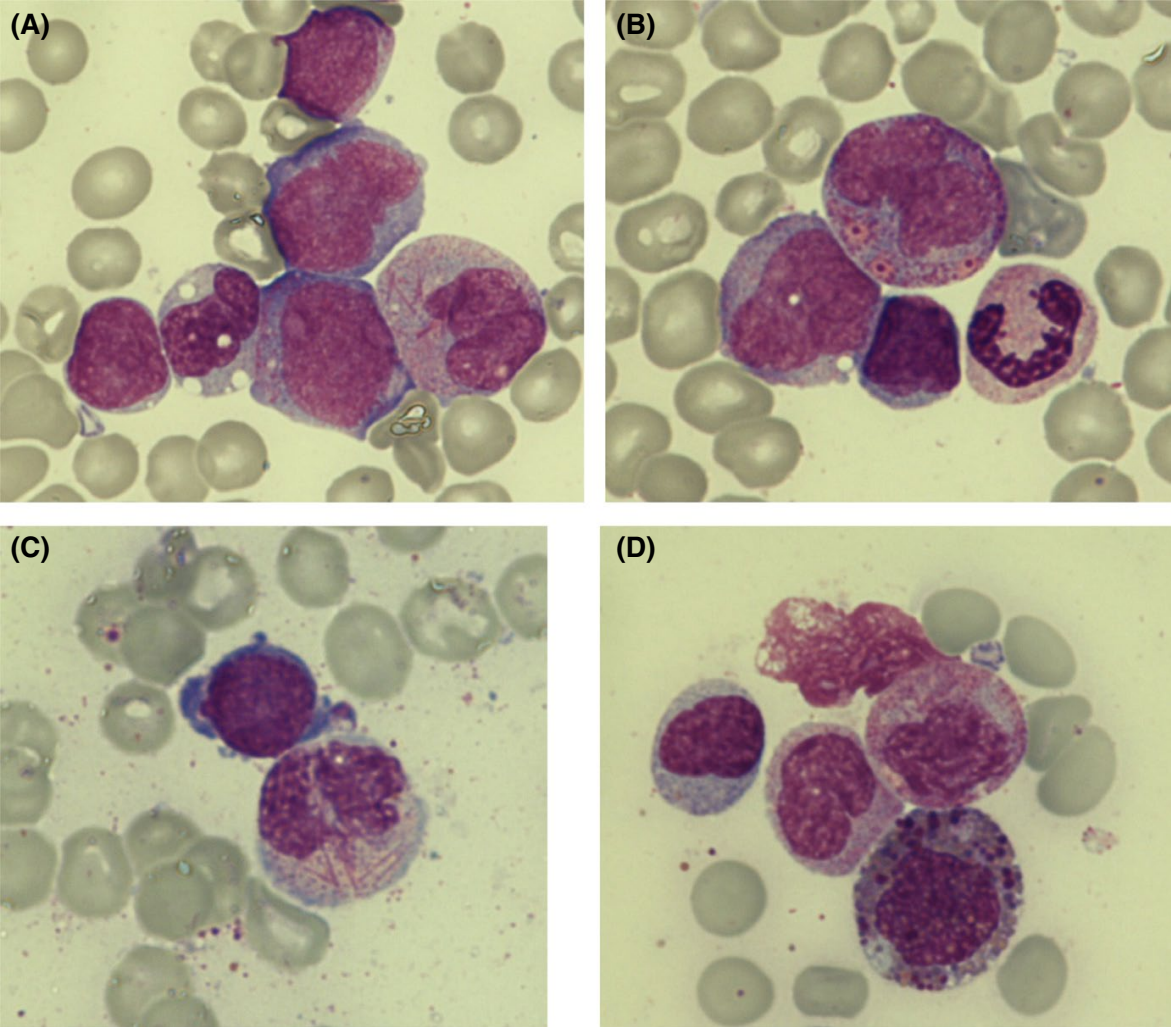
Bone marrow aspirate smear performed at admission (May Grunwald‐Giemsa, 100×). A, Myeloid and monocytoid blasts. A faggot promyelocyte (right). B, Atypical promyelocyte with pseudo‐Chediak‐Higashi granules. C, Dysplastic neutrophil with bundles of Auer rods (Neutrophil faggot cell). D, Pre‐eosinophilic granulation in an eosinophil myelocyte

BM flow cytometry analysis revealed a large blast population with myeloid phenotype and a minor monocytic population. Cytogenetic analysis displayed a normal karyotype. Molecular analysis confirmed inv(16)(*CBFb‐MYH11*) and excluded *PML‐RARA* (t(15;17)). Final diagnosis was therapy‐related acute myeloid leukemia (t‐AML) with inv(16). She received induction and consolidation chemotherapy followed by an allogeneic stem cell transplantation.

Faggot cells are characteristically found in atypical promyelocytes of acute promyelocytic leukemia (APL) but they cannot be regarded as specific.^1^ The presence of faggot cells in non‐APL is rare, whereas the finding of faggot cells in mature granulocytic cells is rarer. Furthermore, it is important to note that inv(16) should be dismissed when atypical pre‐eosinophilic granulation is found. Neutrophil faggot cells are a very uncommon feature described in t‐AML with inv(16),^2^ with a single case reported in literature.^1^

## CONFLICT OF INTEREST

The authors of this paper have no conflicts of interest, including specific financial interests, relationships, and/or affiliations relevant to the subject matter or materials included.

## AUTHOR CONTRIBUTIONS

AVGDV: revised the case, wrote, and edited the manuscript. CDMS: performed morphological examination of the peripheral blood film/bone marrow aspirate, captured the microscopic images, and revised the manuscript. DRDC: performed morphological examination of the peripheral blood film/bone marrow aspirate and revised the manuscript. VRG: helped on morphological examination. AMA: revised the manuscript. JMGDC: was the physician involved in patient´s care.

## Data Availability

Data sharing not applicable to the article as no datasets were generated.
